# Conservation of *Verbascum sinaiticum* Benth using innovative tissue culture technique and DNA barcoding

**DOI:** 10.1038/s41598-025-10968-1

**Published:** 2025-07-18

**Authors:** Mohamed Abdel-Shakur Ali, Nora Rabee Gowda, Mai A. Allam, Sayed A. Fayed

**Affiliations:** 1 Biochemistry Department, Faculty of Agriculture, Cairo University, Giza, Egypt; 2 Plant Biotechnology Department, Biotechnology Research Institute, National Research Centre (NRC), Dokki, 12622 Giza Egypt

**Keywords:** Medicinal plants, Endangered plants, *Verbascum sinaiticum* Benth, DNA barcoding, SDS, HPLC, Biotechnology, Molecular biology, Plant sciences

## Abstract

Egypt has diverse medicinal plants, many of which are endangered because of environmental and human pressures. Verbascum sinaiticum Benth (V. sinaiticum) is a medicinal plant from the Scrophulariaceae family and a locally rare species endemic to Sinai. This study introduces an innovative in vitro regeneration protocol for V. sinaiticum, developed for the first time using optimized tissue culture techniques. Additionally, the incorporation of molecular identification by DNA barcoding and biochemical profiling (via HPLC and SDS-PAGE) presents a complete strategy to conservation and genetic documentation, to support future biotechnological applications. An efficient in vitro regeneration system for V. sinaiticum was established using tissue culture techniques. The study investigated the effects of three different shoot induction media on growth, The first medium was BA medium, a combination of 0.5 mg L^− 1^ benzyl adenine (BA), 5 mg L^− 1^ thiamine HCl, 0.5 mg L^− 1^ nicotinic acid, and 0.5 mg L^− 1^ pyridoxine was added to the initial medium. The second medium was MD medium supplemented with 3 mg L^− 1^ BA, 0.2 mg L^− 1^ naphthaleneacetic acid (NAA), 10 mg L^− 1^ thiamine HCl, 1 mg L^− 1^ nicotinic acid, and 1 mg L^− 1^ pyridoxine. The third medium was AB medium formulated with 1 mg L^− 1^ Adenine sulfate, 1 mg L^− 1^ benzyl adenine, 10 mg L^− 1^ thiamine HCl, 1 mg L^− 1^ nicotinic acid, and 1 mg L^− 1^ pyridoxine, and three media for rooting, The first medium was IB medium supplemented with 0.4 mg L^− 1^ indol butyric acid, 5 mg L^− 1^ thiamine HCl, 0.5 mg L^− 1^ nicotinic acid, and 0.5 mg L^− 1^ pyridoxine. The second medium was IA medium containing 5 mg L^− 1^ thiamine HCl, 0.5 mg L^− 1^ nicotinic acid, and 0.5 mg L^− 1^ pyridoxine. The third medium was NA media formulated with 0.4 mg L^− 1^ naphthaleneacetic acid, 5 mg L^− 1^ thiamine HCl, 0.5 mg L^− 1^ nicotinic acid, and 0.5 mg L^− 1^ pyridoxine. All media were prepared using Murashige and Skoog (MS) basal salts 4.4 g L^− 1^ supplemented with 30 g L^− 1^ sucrose, and solidified with 2.8 g L^− 1^ gelrite. Various propagation protocols were tested based on the availability of plant materials. We have devised flexible methods for large-scale micropropagation that offer a sustainable and quick production strategy, even in the face of resource constraints. The surface sterilization technique was refined, resulting in up to 75% contamination-free cultures. The highest germination rate was observed with 20% Clorox for 15–20 min, but extended exposure (25–30 min) resulted in decreased germination.HPLC analysis revealed that the ethanolic extract had the highest quantities of rutin (19.07 µg/mL) and vanillin (6.29 µg/mL), while the aqueous extract had the highest levels of gallic acid (7.02 µg/mL) and chlorogenic acid (6.02 µg/mL). SDS-PAGE examination revealed five protein bands ranging in molecular weight from 240 kDa to 28 kDa. This study introduces an innovation and preservation strategy for V. sinaiticum, a locally rare species in Egypt. DNA barcoding was used for molecular authentication, and the acquired sequence was matched to GenBank database sequences using the BLAST tool.

## Introduction

The conservation of endangered plant species, especially medicinal plants, is a critical issue because they face significant threats from environmental changes and human activity. The *Verbascum sinaiticum* Benth herb is the tenth member of the Scrophulariaceae family^1^. Locally, it was a rare species in the South Sinai region of Egypt because of increasing pressure due to overgrazing, habitat fragmentation, and limited distribution. Based on records, it was the most popular species for treating asthma and the most popular medicinal plant for lowering blood pressure^2–4^. Its fresh roots are frequently blended with water and used orally, either fresh or dried, and are occasionally swallowed quickly after snake bites^5^. In addition, the roots have long been used in traditional medicine to cure wounds. However, little scientific research has been done on its medicinal qualities or conservation status^6^.

Recent research emphasizes the necessity of preserving Veronica sinaiticum at the local level, with in vitro culture regarded as an effective strategy for the rapid multiplication of threatened plants. Artificial seed germination, seed storage, and greenhouse growing are all preservation procedures that aim to ensure conservation and increase availability for medicinal and biotechnological uses^7,8^. DNA barcoding is an important technique for the conservation of endemic and uncommon plant species, as it allows for precise species identification and genetic documentation^9–13^. The CBOL supports two plastid genes, *rbcL* (ribulose-1,5-bisphosphate carboxylase/oxygenase large subunit) and *matK* (maturase K), as universal barcodes for terrestrial plants^14^. Other interesting barcodes include the plastid intergenic spacer psbA-trnH and the nuclear Internal Transcribed Spacer (ITS), both of which are non-coding areas in their respective genomes^15–17^. The ITS2 region has been proposed as a universal barcode for medicinal herbs, using psbA-trnH as an additional marker^11,18^.

The goal of this work is to create an optimal in vitro propagation method for *V. sinaiticum*. Additionally, DNA barcoding was used to genetically describe *V. sinaiticum*. The acquired sequences were matched to similar sequences from the NCBI database to establish species identity and phylogenetic relationships. These sequences were also submitted to public databases to document and preserve genetic information, which will aid in species discrimination and conservation efforts based on our findings^19^.

## Materials and methods

### Chemicals

The chemicals and reagents utilized in this study were of analytical grade. The following chemicals were used: nicotinic acid, pyridoxine, Gelrite (LOBA), thiamine HCl (Duchefa Biochemie), ethanol (Fisher), butyric acid, benzyl adenine, adenine sulfate, indole-3-acetic acid, naphthaleneacetic acid (Meron), and sucrose (ALDRICH), along with Murashige and Skoog medium (Caisson).

### Plant material

*Verbascum sinaiticum* seeds were collected in North Sinai, Egypt, where they grow naturally. Dr. Omran Ghaly, Director of the Plant Taxonomy Unit at the Desert Research Center (DRC) in Mataria, Cairo, classified the plant species taxonomically. This identification was based on physical characteristics and institutional knowledge. A herbarium voucher specimen was not submitted because the material has been authenticated and documented in DRC records.

### Tissue culture protocol

#### Surface sterilization and seed germination stage

The initial phase of tissue culture involved surface sterilization and germination of seeds. First, seeds were placed in a laminar airflow hood and sterilized with 70% ethanol for 1 min, followed by immersion in sodium hypochlorite solution (Clorox) at varying concentrations (15%, 20%) and exposure times (15,20,25,30 min). After sterilization, the seeds were thoroughly rinsed 3 to 5 times with sterile distilled water and dried using filter paper. Murashige and Skoog (MS) medium^20^ was prepared with 4.4 g L^−1^ and supplemented with 3% sucrose. The pH of the medium was adjusted to 5.7–5.8 using 0.10 N NaOH and 0.10 N HCl. Gelling was achieved by adding 2.8 g L^−1^ of gelrite. Finally, the medium was sterilized by autoclaving at 121 °C for 20 min and incubated for two days to confirm the absence of contamination before cultivating the seeds. Seeds were cultured under sterile conditions in four separate experiments and incubated in the dark until germination occurred. General incubation conditions for all stages included a temperature of 25 ± 2 ºC and a photoperiod of 16 h light/8 h dark, provided by a cool-white fluorescent light lamp Philips TLD 36 W/840 providing approximately 36 W power, 1200–1500 lumens light intensity.

#### Shoot induction and multiplication stage

In this phase, shoots and leaf explants taken from In vitro-germinated *V. sinaiticum* plantlets were cultured on three different media formulation to examined their effects on shoot induction and multiplication. Each medium type was based on full-strength MS basal salts 4.4 g L^−1^ MS, supplemented with 30 g L^−1^ sucrose, and gelling with 2.8 g L^−1^ gelrite. The three media tested were as follow, the first media was BA medium combination of 0.5 mg L^−1^ benzyl adenine (BA), 5 mg L^−1^ thiamine HCl, 0.5 mg L^−1^ nicotinic acid, and 0.5 mg L^−1^ pyridoxine was added to the initial medium. The second media was MD medium supplemented with 3 mg L^−1^ BA, 0.2 mg L^−1^ naphthaleneacetic acid (NAA), 10 mg L^−1^ thiamine HCl, 1 mg L^−1^ nicotinic acid, and 1 mg L^−1^ pyridoxine. The third media was AB medium formulated with 1 mg L^−1^ Adenine sulfate, 1 mg L^−1^ benzyl adenine, 10 mg L^−1^ thiamine HCl, 1 mg L^−1^ nicotinic acid, and 1 mg L^−1^pyridoxine. All media were brought to a pH of 5.7 to 5.8. All cultures were maintained under controlled environmental conditions in the culture room at 25 ± 2 ºC at 16 h light/8 h dark.

#### Rooting stage

The Murashige and Skoog (MS) base salts (4.4 g L⁻¹) were solidified with 2.8 g L⁻¹ gelrite and 30 g L⁻¹ sucrose. The medium (IB) included 5.0 mg L⁻¹ thiamine-HCl, 0.5 mg L⁻¹ nicotinic acid, 0.4 mg L⁻¹ indole-3-butyric acid (IBA), and 0.5 mg L⁻¹ pyridoxine. The second medium (IA) lacked auxin and contained only 0.5 mg L⁻¹ nicotinic acid, 0.5 mg L⁻¹ pyridoxine, and 5 mg L⁻¹ thiamine-HCl. The third medium (NA) received the same vitamin concentrations as the IA medium and was supplemented with 0.4 mg L⁻¹ naphthaleneacetic acid. All media were adjusted to a pH of 5.7 to 5.8, and all cultures were maintained under controlled conditions.

#### Acclimatization of plantlets

After successful rooting, Plantlets were carefully transferred from the culture media containers and washed thoroughly with distilled water to remove any remaining gelling agent and immersion with anti-fungal solution to minimizing the risk of contamination after that the plantlets were transplanted into pots with peat moss and sand (1:1) then covered with clear plastic bags, to maintain high humidity for the first three weeks, they were placed in the sun to ensure hardening. After three months of twice-weekly watering, the plantlets were transferred into the soil.

### Biochemical characterization

#### HPLC of bioactive compounds

Fresh plant shoots were weighed, chopped, and ground using a mortar and pestle in a hydroethanolic solvent mixture (H_2_O–ethanol). The homogenized mixture was then transferred to a clean flask and kept on a shaker overnight at 25 °C for complete extraction. Then the mixture was filtered with medical gauze to remove the solid material from the extract. The resulting filtrate was treated with half the volume of methylene chloride to eliminate lipids and chlorophyll. The organic (methylene chloride) phase containing lipophilic impurities was discarded. The final extract Should Be Kept at 4 °C and 20 °C until further biochemical analysis^21^.

The Agilent 1260 series analytical HPLC system was used to determine the phenolic profile at the National Research Center (NRC), Dokki, Egypt. The separation was performed using an Eclipse C18 column (4.6 mm x 250 mm i.d., 5 μm particle size). The mobile phase consisted of water (A) and 0.05% trifluoroacetic acid in acetonitrile (B) at a flow rate of 0.9 ml/min. The sequential linear gradient programming for the mobile phase is as follows: 8 to 12 min (60% A); 15 to 15 min (82% A); 15 to 16 min (82% A); and 16 to 20 min (82% A). 280 nm was the monitoring wavelength for the multi-wavelength detector. Five microliters (µL) were the injection volume for every sample solution. At 40 °C, the column temperature was kept constant.

#### SDS-PAGE protein profiling

Samples were weighed using fresh shoots, pulverized in a mortar with liquid nitrogen, and then combined with phosphate buffer (0.02 M, pH 7) as an extraction buffer at a volume of 1:2. the mixture was centrifuged at 12,000 rpm for 15 min. For protein precipitation, another tube was used to hold the supernatant, with two milliliters of TCA (10%) in acetone and two ME (0.07%). After 15 min of centrifugation at 13,000 rpm and 4 °C, the protein pellet was produced. Three rounds of washing with cooled acetone supplemented with two ME (0.07%), two milligrams of EDTA, and one tablet of a complete EDTA-free protease inhibitor were used to purify the pellet. Pure acetone was used during the last washing. Overnight, the pellet was stored at −80 °C. After dissolving in a rehydration buffer, the acetone-free pellet was measured.

The final concentrations in the extraction buffer were Tris-HCl 0.5 M to adjust (pH 6.8), SDS 2.5%, glycerol 10%, and 2-mercaptoethanol 5%. To follow the mobility of the protein in the gel, bromophenol blue (BPB) was added to the sample buffer. Protein was examined using slab-type SDS-PAGE on an 11.25% polyacrylamide gel. Two different gels were performed under comparable electrophoretic conditions to verify the method’s repeatability. Utilizing the “MW-SDS-70 kit”, a molecular weight protein standard, the molecular weights of the dissociated polypeptides were ascertained. Before creating a workable solution, stock solutions were made using all of the ingredients that were acquired from Sigma Chemical Company in the USA. A Flourchem computer program and a gel documentation system were used to take pictures of the gels. The total protein SDS-PAGE was performed in the discontinuous buffer system using the technique of^22^.

### DNA barcoding

#### DNA extraction, amplification, and sequencing

Total genomic DNA was isolated from dried leaves taken from in vitro-derived tissues using the manufacturer protocol from the Qiagen DNeasy for Plant mini kit (Qiagen, Germany). The standard plant barcodes, *matK* and *rbcL*, which are specific regions of two plastids, were amplified separately using a Biometra Tone 96G thermal cycler (Analytik Jena GmbH, Germany), For the 25 µL total volume PCR reactions, the following ingredients were utilized: Flexi DNA polymerase GoTaq^®^ G2 (Promega, USA). The final volume was supplemented with 1.25 units, 0.2 mM dNTPs, 2.5 mM MgCl_2_ (Promega, USA), 50–80 ng of template DNA, 10 pmol of each primer (Macrogen Inc., South Korea), and nuclease-free water. The *matK* and *rbcL* barcode regions were amplified by PCR using the subsequent thermocycling; after examining and photographing the gels, the PCR results were compared to a 100 bp DNA ladder to evaluate their size. (Genedirex, Taiwan) (Fig. 5). As directed by the manufacturer, the amplified products were purified using the Promega Wizard SV Gel and PCR cleaning supplies (Promega, USA). At Macrogen Inc. in South Korea, the enhanced PCR products underwent bidirectional sequencing. The same primers of PCR have been used in the sequencing procedure (Table 1). After sequencing, the forward and reverse trace files’ low-quality ends were removed, and solid sequences free of gaps or stop codons were created using CodonCode Aligner v9.0.2 (CodonCode Co., USA). The final sequences were submitted to the NCBI GenBank database, and accession numbers were obtained^23^.

**Table 1:** PCR amplification of the two primer pairs, *MatK* and *RbcL* regions^23^.

Loci	Primer	Sequence (5’->3’)	Product size(bp)	Primer concentration (pmol/uL)	Reference
*matK*	*matK*−390 F	CGATCTATTCATTCAATATTTC	600 bp Over	10	^24^
	*matK*−1326R	TCTAGCACACGAAAGTCGAAGT			
*rbcL*	*rbcL*−1 F	ATGTCACCACAAACAGAAAC	600 bp Over	10	^14,25^
	*rbcL*−724R	TCGCATGTACCTGCAGTAGC			

#### DNA barcoding analysis

To evaluate the efficiency of DNA barcoding in identifying the examined specimens at the family, genus, and species levels, plant barcode sequences (*matK* and *rbcL*) were evaluated against the GenBank database using the Basic Local Alignment Tool (BLAST). The top five alignment hits with the greatest bit-scores and lowest E-values were selected for the subsequent phylogenetic analysis. Plant sequences with mismatched alignment or those that did not belong to the right genus or species were not included in the phylogenetic analysis. Models that score the lowest on the Bayesian Information Criterion (BIC) are the ones that describe nucleotide substitution patterns, which are determined by identifying the best-fitting model estimate. Additionally included for each model are the AICc value (Akaike Information Criterion, corrected) and the number of parameters (including branch lengths)^26^. The non-uniformity of rates of evolution at different sites may be simulated by using five scales that represent major categories present in a separate Gamma dispersion (+ G) and assuming that there are a specific percentage of evolutionarily invariant sites (I), Estimates of the percentage of invariant sites and/or the gamma shape parameter are displayed where appropriate. Additionally displayed for every model are the transition/transversion bias (R) Figures, whether it is assumed or calculated. Nucleotide frequencies (f) and base substitution rates (r) follow each nucleotide pair. When assessing them, one should take into account the respective values of instantaneous r. For the sake of simplicity, the sum of each model’s r values is set at 1. To determine ML values, an automatic tree topology was developed. The tree was constructed using MEGA11 with 500 bootstrap replicates. Bootstrap values (BS) are shown at the nodes to indicate the level of support for each clade. In this study, four Sequences of nucleotides were examined. Less than 5% of alignment spaces, missing data, and ambiguous bases were permitted at any position, and all sites with less than 95% site coverage were removed (Option for partial deletion). In all, 621 places were included in the final dataset. Making use of MEGA11^27^, in addition to performing evolutionary analysis, the tree topology was automatically calculated. Four nucleotide sequences were used in this study. Posts that were fewer than 95% of the sites that were covered were removed, while posts with fewer than 5% alignment spaces, missing data, and unclear bases were allowed anywhere (option for partial removal). In all, 621 places were included in the final dataset. Evolutionary analysis was performed with MEGA11 (Molecular Evolutionary Genetics Analysis Version 11), molecular evolution, as well as phylogenetic^27–30^.

### Data analysis and software

All analyses and data processing were conducted using software programs specialized for each methodological stage of this study.

#### Statistical analysis of in vitro culture data

Two-way ANOVA was used to analyze germination data affected by exposure time, sterilization concentration, and their interaction. The analysis was conducted using Python (version 3.x). The null hypothesis (H₀) was rejected for the exposure time, with a *p-value* of 0.0098, and for the interaction between concentration and duration, as p-values were below 0.05, indicating statistically significant differences^31^.

#### SDS-PAGE, DNA barcoding, and phylogenetic data

Protein band patterns of SDS-PAGE were analyzed by GelAnalyzer v19.1. Chromatograms of DNA sequences were edited and assembled by CodonCode Aligner v9.0.2 (CodonCode Co., USA). The amplified sequences were identified by the BLAST tool that searched against the NCBI GenBank database. Phylogenetic analysis and a tree were conducted using MEGA 11 software.

## Results and discussion

### Tissue culture

#### Surface sterilization and seed germination

The in vitro seed germination of *V. sinaiticum* was assessed using various sterilization techniques. The average rate of microbial contamination was about 25%. Under optimal sterilization conditions, the maximum germination rate in contamination-free cultures reached 75%. Seedlings produced under these conditions displayed elongated stems, closely spaced nodes, and numerous leaves; however, overall growth remained limited. To promote propagation, leaf segments from sterilized, germinated seedlings were cultured in full-strength MS media supplemented with various concentrations of BA, AS, and vitamins. After five weeks of cultivation, significant variation was observed in both leaf number and shoot length among treatments. Statistical analysis using ANOVA presented in Table 2 revealed significant differences between treatment groups. The highest average germination was noted with 20% Clorox for 15–20 min (12.25 ± 0.35), followed by 15% for the same duration (10.62 ± 0.88). In contrast, extended sterilization times of 25–30 min, especially with 20% Clorox, resulted in reduced germination (2.38 ± 0.88). Thus, the germination rates were negatively affected by an increased sterilization period.

**Table 2:** Effect of concentrations and durations of sodium hypochlorite on seed germination.

Plant	Treatments		Germination %
	Clorox conc (v/v)	Duration (min)	
*V.sinaiticum*	15	15–20	10.62 ± 0.88^a^
	15	25–30	4.00 ± 3.18^b^
	20	15–20	12.25 ± 0.35^c^
	20	20–30	2.38 ± 0.88^d^

Each value represents the mean ± standard deviation, *n* = 3. Treatments followed by the same letter do not differ.

#### Shoot induction and multiplication

**Fig. 1:**
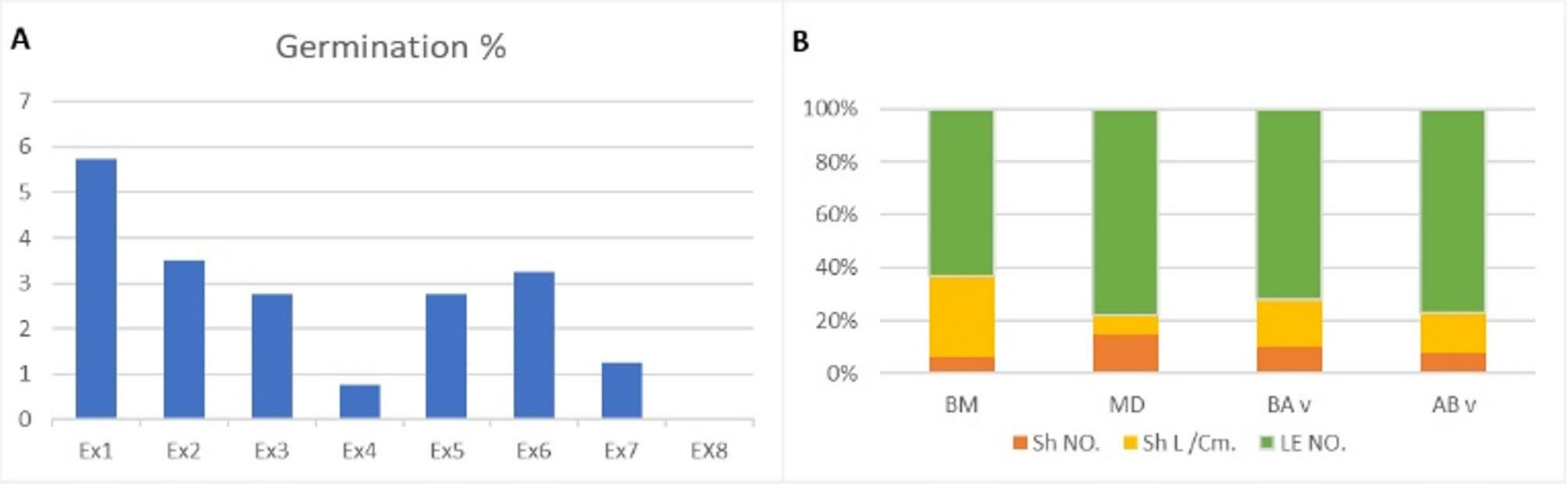
(**A**) Total germination at different durations and concentrations of Clorox on BM media. (**B**) compare leaves number, shoot length, and shoot number between control media BM and three types of shooting media MD, BA, AB.

The effects of three different shoot induction media- MD, BA, and AB- were evaluated after eight weeks of culture, as illustrated in Fig. 1 (A). The parameters assessed included the number of leaves, shoot length, and number of shoots per explant. The MD medium, which contained benzyl adenine and naphthaleneacetic acid, resulted in the highest values for measured traits. Specifically, the MD medium produced average values of 5 shoots, 6 cm shoot length, and 18 leaves per explant Fig. 1 (B). Explants cultured on MD also exhibited the highest number of axillary shoots and plantlets, followed by those grown on AB medium (containing adenine sulfate and benzyl adenine). In contrast, the BA medium alone (without NAA or AS) yielded fewer shoots and shorter shoot lengths, indicating reduced efficiency in shoot proliferation. The results are consistent with previous studies by Hassan^32^ and Aggarwal and Barna^33^, which also reported enhanced shoot multiplication under similar hormonal treatments. Measurements recorded for mother plants cultured in BM medium remained stable across all parameters.

#### Rooting and acclimatization of plantlets

**Fig. 2:**
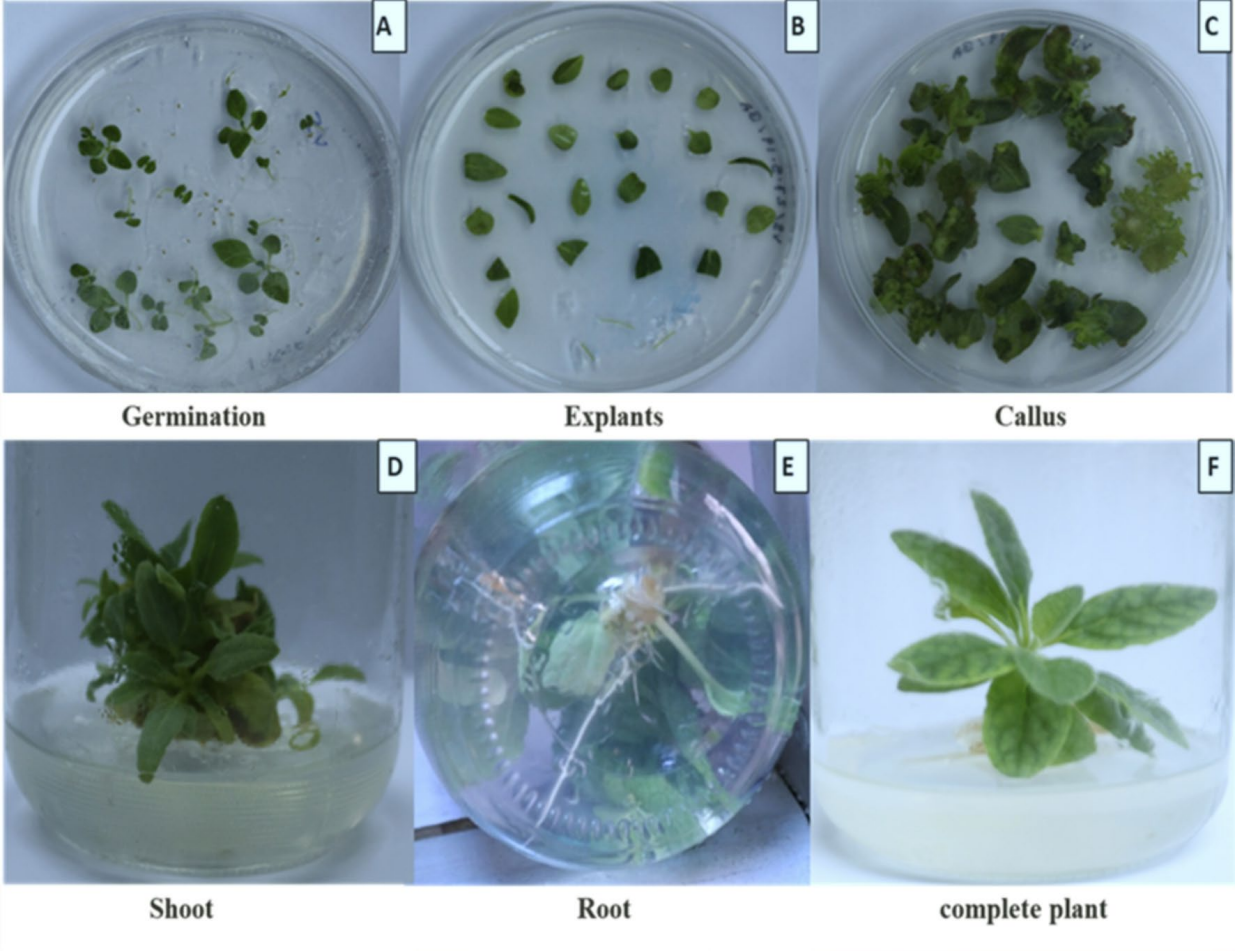
In vitro plant micropropagation protocol of Verbascum sinaiticum Benth.: (**a**) aseptic seedling; (**b**) leaves cutting as explants; (**c**) callus formation; (**d**) shoot formation on MD medium; (**e**) roots formed on MS medium fortified with 0.4 mg/L IB (indole-3-butyric acid); (**f**) complete plant on MS medium.

**Fig. 3:**
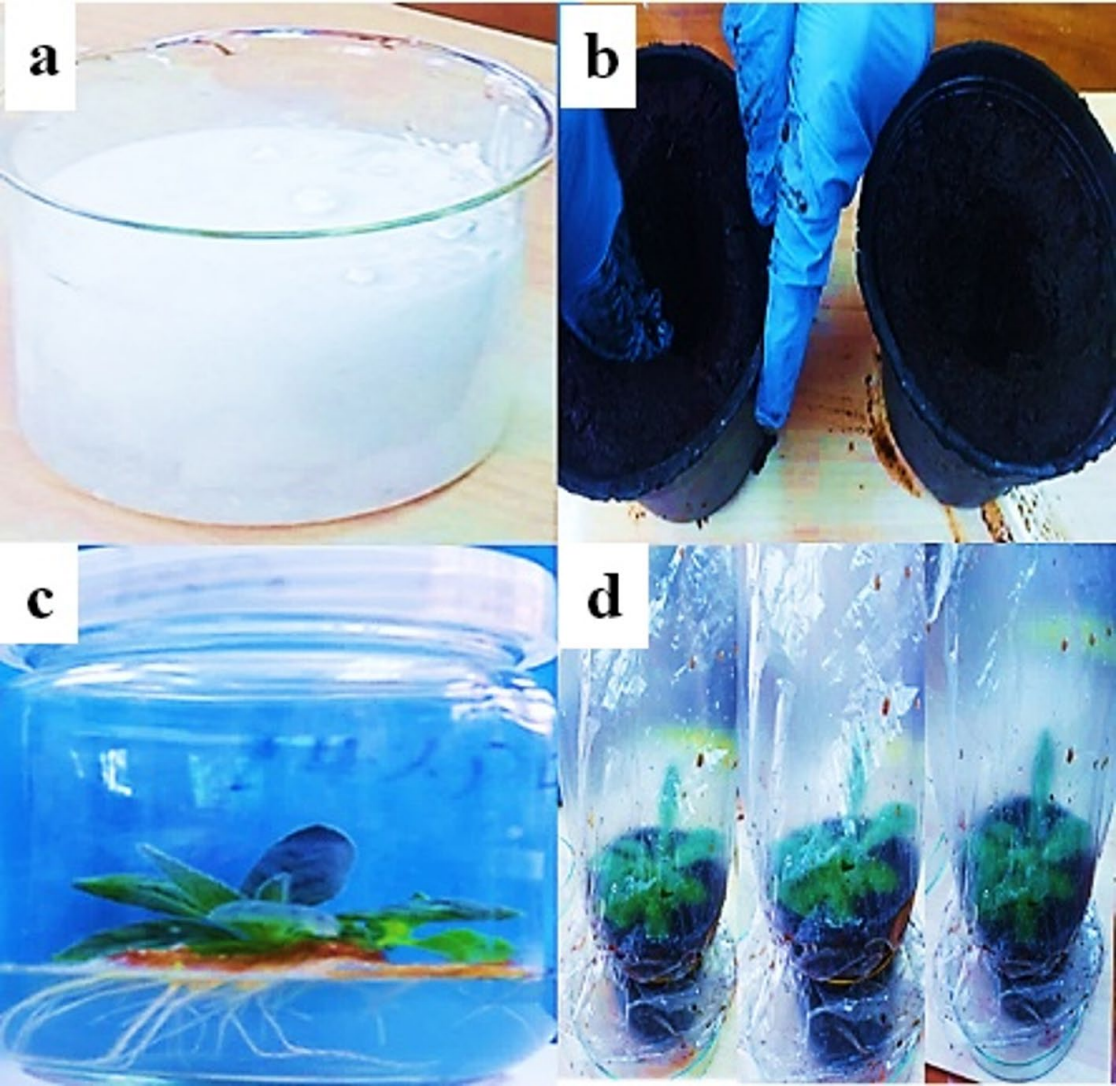
In vitro rooting and acclimatization of Verbascum sinaiticum Benth plantlets; (**A**) anti-fungus solution (**B**) acclimatization Preparation (**C**) In vitro rooting on MS basal medium with IB. (**D**) Acclimatization of the in vitro-rooted plantlets under plastic bags.

Root induction was assessed using full-strength MS media supplemented with different types and concentrations of auxins, including IBA, IAA, and NAA. After eight weeks, all treatments resulted in root formation, but with significant differences in root number and length depending on the auxin type. Among the auxins tested, IBA produced the most robust rooting responses, with longer and more numerous roots compared to IAA and NAA, as shown in Figs. 2 and 3. Nevertheless, IAA and NAA also induced rooting, but the resulting roots were shorter and less vigorous. The data clearly show that combining MS medium with IBA yielded the most effective and consistent root systems across all measured parameters. These findings are consistent with previous studies^32,34–36^, which reported similar outcomes in other plant species regarding the superior rooting effects of IBA.

### Biochemical characterization

#### HPLC of bioactive compounds

The HPLC analysis of the phenolic compounds in the ethanolic (Et) and aqueous (Aq) extracts of *V. sinaiticum* shoots revealed 14 chemicals in the Et extract and 14 in the Aq extract, with the quantities of these compounds varying between samples (Table 3). The Et extract contained the highest concentrations of Rutin (19.07 µg/mL) and Vanillin (6.29 µg/mL), along with Catechin, Syringic acid, and Hesperetin (3.63, 3.06, and 0.31 µg/mL), which were absent in the Aq extract. These findings contradict those of Kızıltaş et al.^37^ for Verbascum speciosum. The Aq extract exhibited the highest concentration of gallic acid (7.02 µg/mL) and chlorogenic acid (6.02 µg/mL), consistent with Kızıltaş et al.^37^ in Verbascum speciosum. The Et extract also had a notable concentration of ellagic acid (8.22 µg/mL Et and 7.97 µg/mL Et extract), consistent with Çiğdem Aydin et al.^38^ in Verbascum glomeratum. These components have demonstrated various therapeutic characteristics, including anti-inflammatory, antioxidant, and anti-tumor properties, as well as therapeutic effects on metabolic, cardiovascular, cognitive, and gastrointestinal illnesses^39–42^.

**Table 3:** HPLC analysis of phenolic compounds in ethanolic and aqueous extracts of the shoots in *V. sinaiticum*.

NO	Components	Standard		V.sinaiticum	
		µg/mL	Area	Et	Aq
1	Gallic acid	15	190.53	0.26	7.02
2	Chlorogenic acid	50	403.90	1.46	6.02
3	Catechin	75	330.92	3.63	ND
4	Methyl gallate	15	264.26	0.54	0.27
5	Coffeic acid	18	220.07	0.15	1.07
6	Syringic acid	17.2	188.34	3.06	ND
7	Pyro catechol	40	292.50	4.68	2.34
8	Rutin	26	203.95	19.07	0.50
9	Ellagic acid	120	434.15	8.22	7.97
10	Coumaric acid	20	640.01	3.43	0.36
11	Vanillin	12.9	282.68	6.29	0.14
12	Ferulic acid	20	283.37	0.77	0.08
13	Naringenin	30	274.83	3.21	0.12
14	Daidzein	35	541.09	ND	0.07
15	Querectin	40	327.52	ND	0.56
16	Cinnamic acid	10	493.68	ND	0.04
17	Apigenin	50	786.57	ND	ND
18	Kaempferol	30	174.47	ND	ND
19	Hesperetin	20	345.78	0.31	ND

#### SDS-PAGE

**Fig. 4:**
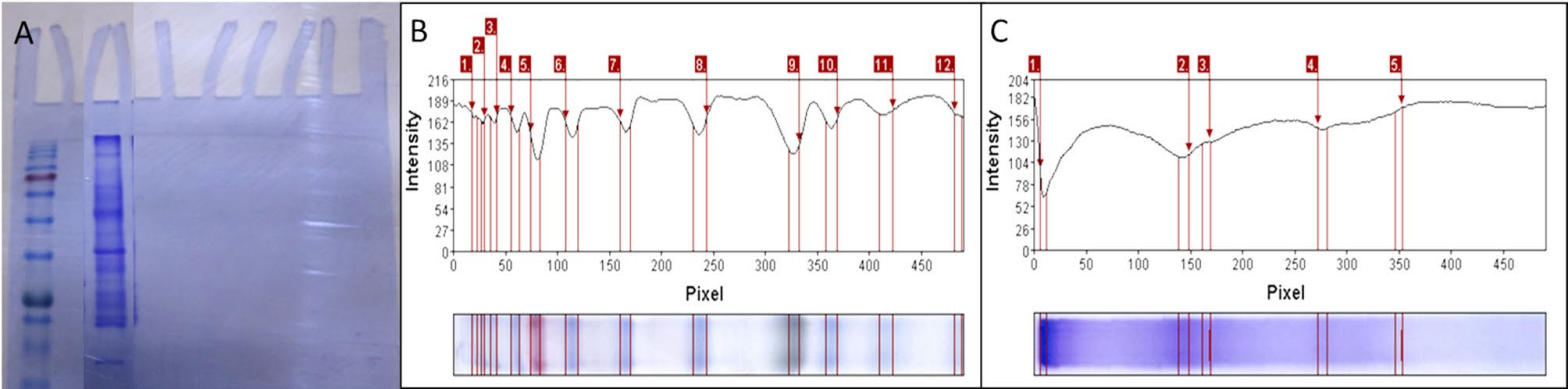
(**A**) Coomassie blue stained SDS-PAGE gel of total protein extracted from micro-propagated plant, first lane, Marker, second lane, V.sinaiticum.(**B**) SDS-PAGE analysis for lane 1, Marker show number of bands 12, M. wt. from 240 to 7 KDa.(**C**) SDS-PAGE analysis for lane 2, V.sinaiticum show number of bands 5, M. wt. from 240 to 28 KDa.

This is the first investigation of SDS-PAGE in *V. sinaiticum*. The entire protein-extracted SDS-PAGE gel was stained with Coomassie blue, as indicated by the marker in lane one, with a molecular weight range of 240 to 7 kDa over 12 bands. *V. sinaiticum* was found in Lane 2, where five protein bands were visible (Fig. 4). Lane 2 comprised bands varying in molecular weight from 240 kDa to 28 kDa, as well as various raw quantities. Band 1 had the lowest molecular weight (240 kDa), while band 4 had the highest (45 kDa). The staining intensity varied from 78 to 182, and the five bands were identified as *V. sinaiticum* species bands. Each of these bands has unique characteristics. The total soluble protein cluster analysis validated the investigation’s discriminability. *V. sinaiticum* has been micropropagated^41,43^.

### DNA barcoding

#### Species identification

**Fig. 5:**
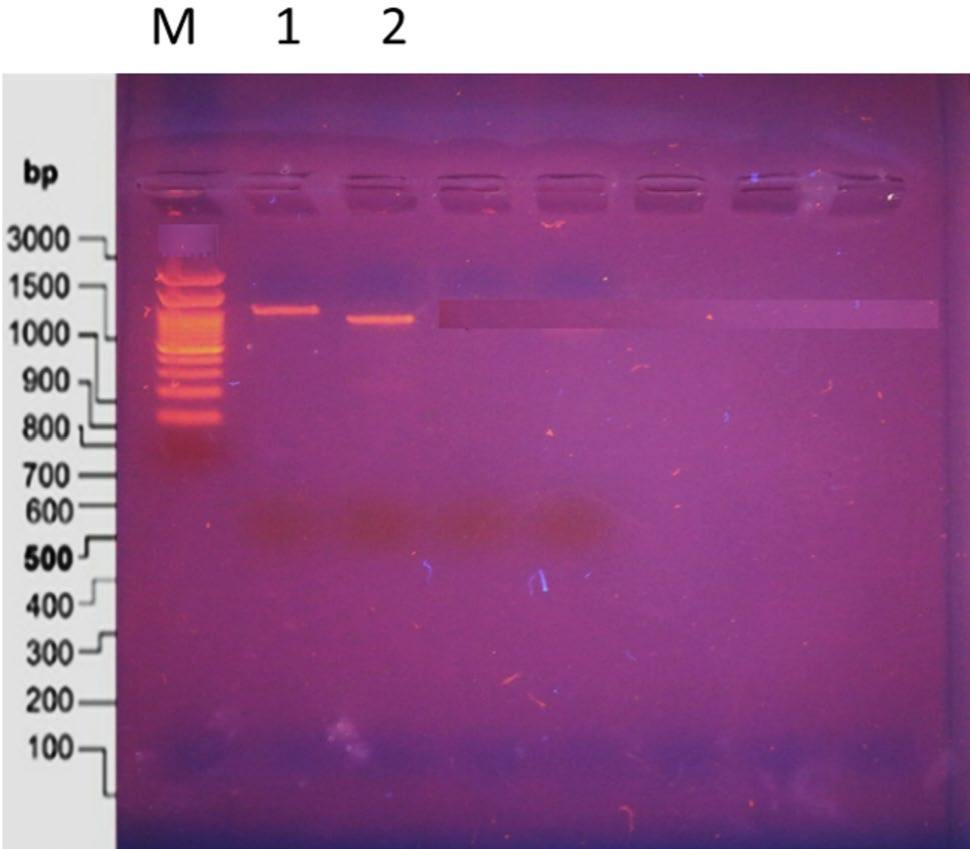
Agarose gel electrophoresis for amplified samples by using the primer matK for sample 1 and rbcL for sample 2.

Successful PCR amplification of the *matK* and *rbcL* barcodes for the *V. sinaiticum* plant produced distinct single bands suitable for accurate DNA sequencing. The amplified sequence lengths of the *rbcL* barcode ranged from 1000 to 900 bp, whereas the matK barcode ranged from 3000 to 1500 bp (Fig. 5). *matK* and rbcL have assembled and aligned sequence lengths of 859 and 666 bp, respectively. The BLAST analysis confirmed that the outcome was successful. A good DNA barcode must have strong discriminatory power, longevity, cost-effectiveness, and global applicability. The two plastid genes, *matK* and *rbcL*, should be the common DNA regions for plant DNA barcoding, according to the Working Committee on CBOL Plants. The findings show that the alignment rates of *V.sinaiticum matK* were 98%, with a bits score of 1587(859), Identities 859/889(100%), gaps 0/859 (0%), and strand plus/plus displayed in Table 4. For the right genus and species of the tested plants as listed in GenBank, the alignment rates of *V.sinaiticum rbcL* are as follows: bits score 1230 (666), Identities: 666/666(100%), Gaps: 0/666(0%), and Strand Plus/Plus are displayed in Table 4. The rate of alignment between the E value in *matK* and *rbcL* was 0.0.

**Table 4:** Maturase K *(matK*) gene partial cds Chloroplast and ribulose-15-bisphosphate carboxylase/oxygenase large subunit (*rbcL*) DNA barcode as downloaded from the NCBI GenBank database.

Gene	Scientific Name	Accession	Query Cover	E value	Identity %
*matK*	*Verbascum sinaiticum*	PP726923.1	98%	0	100
	*Verbascum kermanense*	MH885331.1	99%	0	99.31
	*Verbascum brevipedicellatum*	NC 064357.1	100%	0	98.85
	*Verbascum phoeniceum*	ON121986.1	100%	0	98.62
	*Verbascum nigrum*	JN896238.1	99%	0	99.08
	*Verbascum thapsus*	JN893995.1	99%	0	99.31
*rbcL*	*Verbascum sinaiticum*	PP737189.1	100%	0	100
	*Verbascum songaricum*	NC 085506.1	100%	0	100
	*Verbascum thapsus*	MN192700.1	100%	0	100
	*Verbascum chaixii*	NC 085505.1	100%	0	100
	*Verbascum virgatum*	KM361030.1	100%	0	100
	*Verbascum blattaria*	NC 085504.1	100%	0	99.85

#### Phylogenetic relationship

**Fig. 6:**
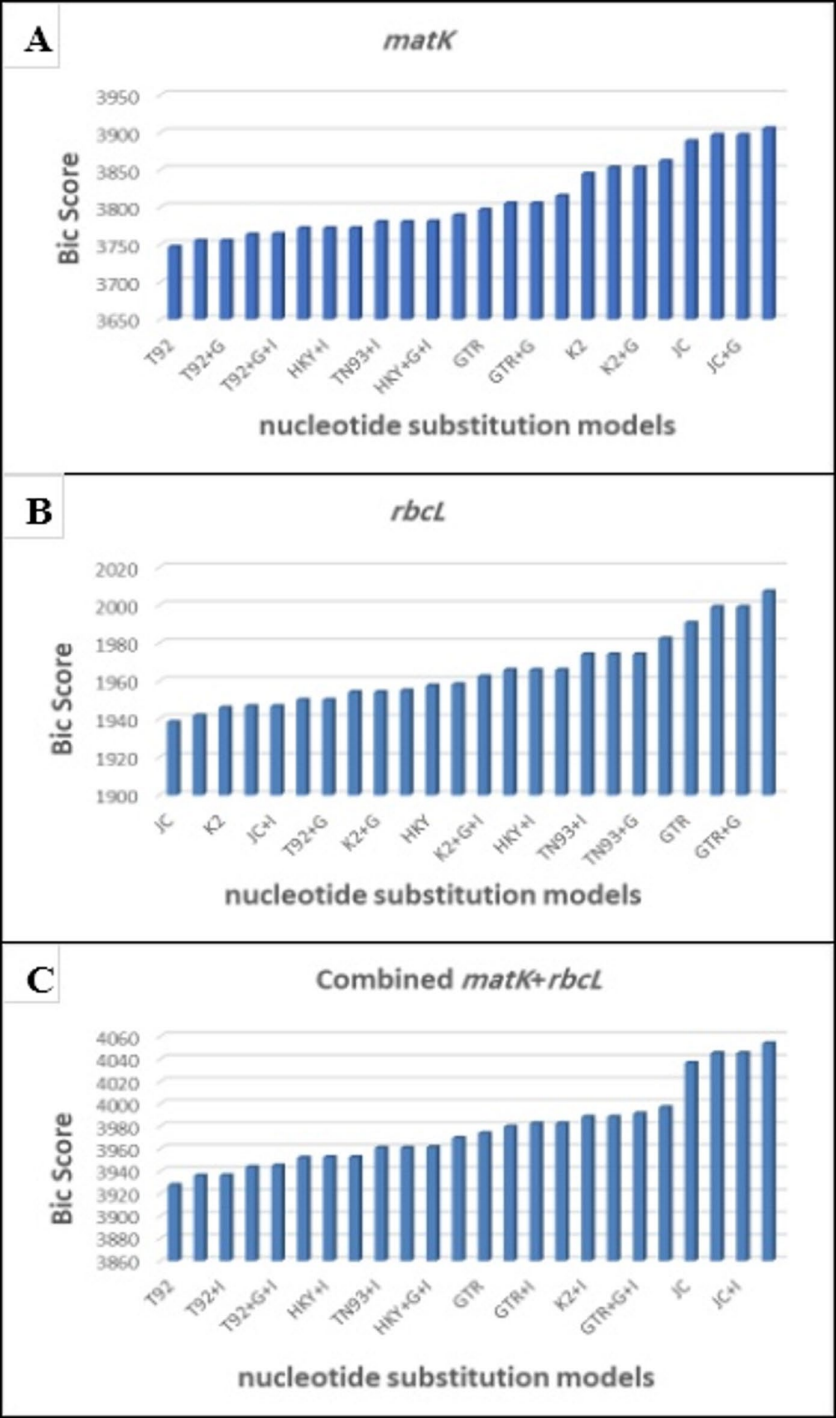
(**A**) Maximum Likelihood fits of 24 different nucleotide substitution models for matK sequences according to BIC scoring. (**B**) Maximum Likelihood fits of 24 different nucleotide substitution models for rbcL sequences according to BIC scoring. (**C**) Maximum Likelihood fits of 24 different nucleotide substitution models for the combined matK+rbcL sequences according to BIC scoring.

It was determined that the Tamura 3-parameter (T92) model was the best machine-learning model for *matK* and the combined *matK* + *rbcL* sequences based on the BIC score, as shown in Fig. 6 (A) and (C). The Jukes-Cantor (JC) model was shown to be the best machine learning model for *rbcL* in Fig. 6 (B), and it is frequently used as a monophyly-based species delimitation technique.

**Fig. 7:**
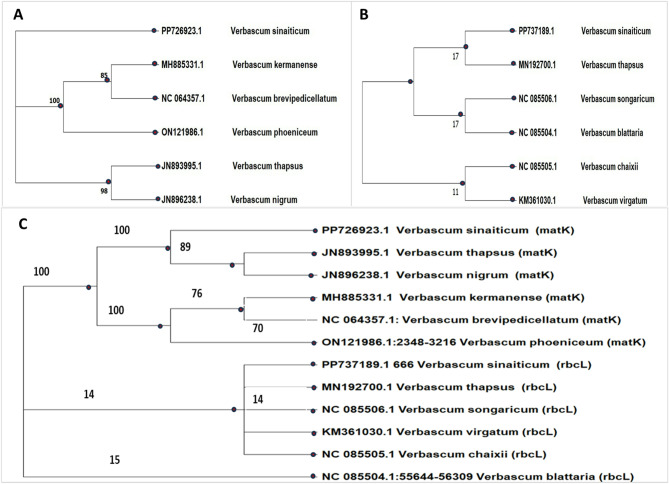
Phylogenetic tree obtained from ML analysis based on (**A**) matK, (**B**) rbcL, and (**C**) combined matK+rbcL DNA barcode sequences. The tree was constructed using MEGA11 with 500 bootstrap replicates. Bootstrap values (BS) are shown at the nodes to indicate the level of support for each clade. The red dots represent the taxa being examined in the current study, and they were aligned against the top 5 sequences retrieved from NCBI GenBank database and are labeled with their corresponding accession numbers.

It is believed that models that have a better representation of the replacement pattern is provided by the lowest Bayesian Information Criterion (BIC) scores. Both the number of parameters and the Maximum Likelihood value (lnL) (including branch lengths), and the AICc value (Akaike Information Criterion, corrected) are also displayed for each model^44^. With five rate categories and the presumption that a specific percentage of sites are evolutionarily invariant (+ I), a discrete Gamma distribution (+ G) can be used to simulate the non-uniformity of evolutionary rates across sites where available, estimations of the predicted percentage of invariant sites and/or the gamma shape parameter do not display. Each model also includes assumed or estimated transition/transversion bias (R) values. The nucleotide frequencies (f) come after them, and base substitution rates (r) for each nucleotide pair. When analyzing instantaneous r, it is important to examine its relative values. For simplicity, the sum of r values is set to one for each model. A tree topology was generated automatically to estimate ML values. This investigation included six nucleotide sequences.

There were three noncoding codon positions listed: first, second, and third. Positions with less than 95% site coverage were eliminated; a partial deletion option allowed alignment gaps, missing data, and ambiguous bases at any position with less than 5%. The completed dataset included a total of 666 locations. MEGA11 was used to undertake evolutionary analysis^39^.

The Phylogenetic tree for the *rbcL* construction method with the (JC) model is shown in Fig. 7 (B), while the ML tree-building method with the (T92) model fundamentally demonstrated a similar topology, notably well-separated from one another inside the tree, Fig. 7 (A) and (C), for *matK* and combined *matK* + *rbcL*. Phylogenetic trees were produced for *matK*,* rbcL*, and the combined tree, the three sequences under study.

## Conclusion

This work proposes an innovative and integrative approach to the conservation and characterization of Egypt’s uncommon and endangered plant species, *V. sinaiticum*. This species was studied for the first time using optimal tissue culture techniques, DNA barcoding, SDS-PAGE protein profiling, and HPLC phytochemical analysis. Despite the scarcity of plant material, the devised in vitro propagation technology allowed for efficient regeneration with minimal space and time constraints. DNA barcoding with *matK* and *rbcL* markers confirmed species identity, and the sequences were submitted to the NCBI database (accession numbers: PP726923.1 and PP737189.1). HPLC examination revealed 14 phenolic and flavonoid components, with ethanolic extracts high in rutin, ellagic acid, and vanillin, and aqueous extracts high in gallic acid, chlorogenic acid, and ellagic acid—compounds recognized for anti-inflammatory, antioxidant, and anticancer activities. SDS-PAGE revealed five different protein bands, and cluster analysis validated the regenerated plants’ biochemical identity. This study provides the first comprehensive biotechnological foundation for preserving *V. sinaiticum* while simultaneously emphasizing its therapeutic potential. The findings provide a solid platform for future conservation measures and drug discovery studies on this unique species.

## Data Availability

The datasets generated and/or analysed during the current study are available in NCBI GenBank database.Verbascum sinaiticum matK gene, partial cds and chloroplast: https://www.ncbi.nlm.nih.gov/nuccore/2724807014?report=graph&tracks=[key:sequence_track,name:Sequence,display_name:Sequence,id:STD649220238,annots:Sequence,ShowLabel:false,ColorGaps:false,shown:true,order:1][key:gene_model_track,name:Genes,display_name:Genes,id:STD3194982005,annots:Unnamed,Options:ShowAll,CDSProductFeats:false,NtRuler:true,AaRuler:true,HighlightMode:2,ShowLabel:true,shown:true,order:4]&v=1:859&c=FF00FF&select=null&slim=0Verbascum sinaiticum rbcL gene, partial cds and chloroplast: https://www.ncbi.nlm.nih.gov/nuccore/2725915198?report=graph&tracks=[key:sequence_track,name:Sequence,display_name:Sequence,id:STD649220238,annots:Sequence,ShowLabel:false,ColorGaps:false,shown:true,order:1][key:gene_model_track,name:Genes,display_name:Genes,id:STD3194982005,annots:Unnamed,Options:ShowAll,CDSProductFeats:false,NtRuler:true,AaRuler:true,HighlightMode:2,ShowLabel:true,shown:true,order:4]&v=1:666&c=FFCC99&select=null&slim=0.

## Abbreviations

rbc: Verbascum sinaiticum Benth
MAPs: Medicinal and Aromatic plants
SKP: Saint Katherine Protectorate
IUCN Red List: International Union for Conservation of Nature
rbcL: ribulose-1, 5-bisphosphate carboxylase/oxygenase large subunit
matK: Maturase K
CBOL: Consortium for the Barcode of Life
psbA–trnH: Plastid photosystem II protein D1 intergenic spacer region
ITS: Internal Transcribed Spacer
BLAST: Basic Local Alignment Tool
BIC: Bayesian Information Criterion
BPB: Bromophenol blue
BA: Benzyl Adenine
NAA: Naphthaleneacetic acid
AS: Adenine sulfate
IBA: Indol Butyric acid
(+ G): Gamma dispersion
TR: General time reversible
HKY: Hasegawa-Kishino-Yano
TN93: Tamura-Nei
T92: Tamura 3-parameter
K2: Kimura 2-parameter
JC: Jukes-Cantor
AICc: Akaike information criterion, corrected
